# Supplementary material for: Influence of Benzothiadiazole on the Accumulation and Metabolism of C6 Compounds in Cabernet Gernischt Grapes (Vitis vinifera L.)
Source: Foods. 2023 Oct 9;12(19):3710. doi: 10.3390/foods12193710 (PMC10572586; doi:10.3390/foods12193710)
Supplement: Supplementary file 1 [file foods-12-03710-s001.zip › Supplementary Materials.pdf]

Figure S1. Changes in temperature and rainfall in Lanzhou City in 2022

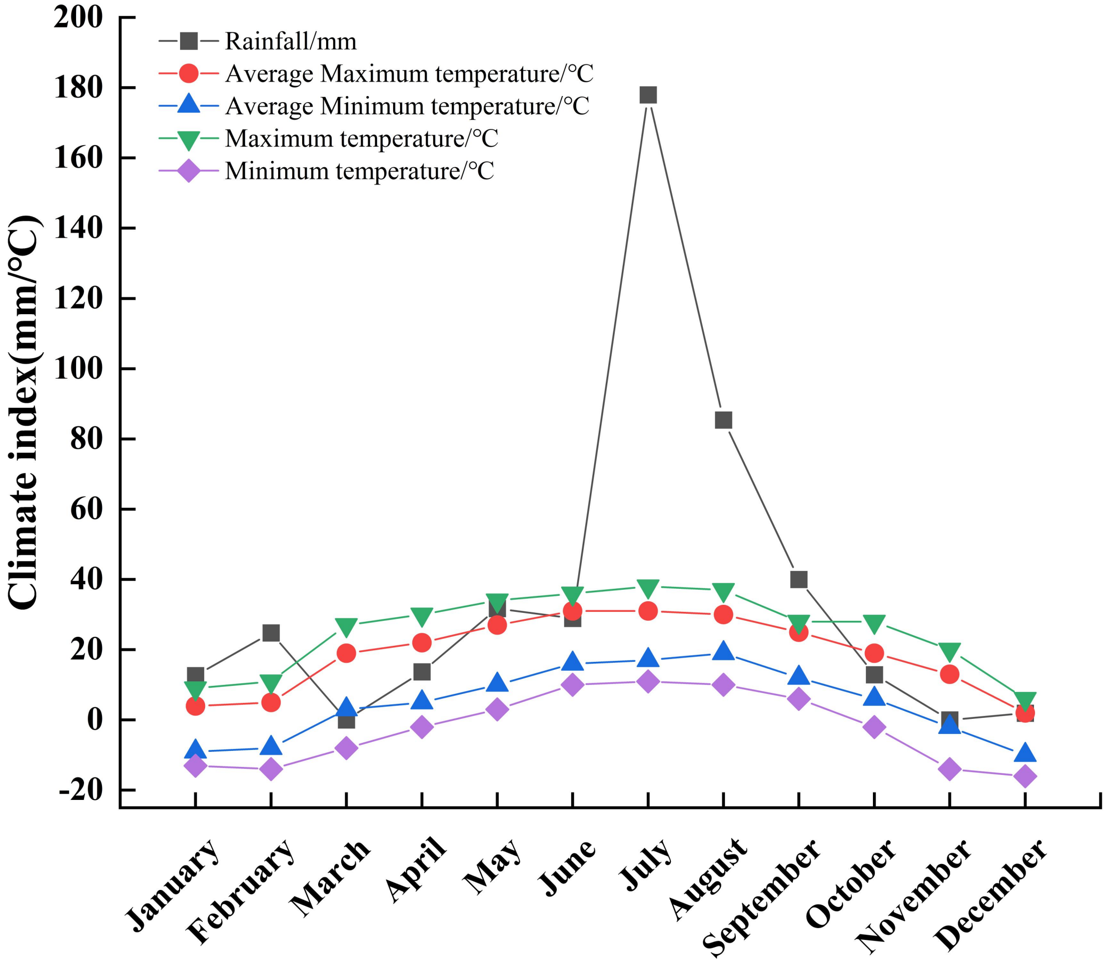

Table S1. Sampling information during Cabernet Gernischt grapes' ripening

Sampling date	Weeks post-flowering	Development stages	Sample description	Sample images
17 June 2022	3	Pre-swelling	Green berries were still hard	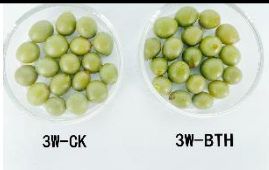
1 July 2022	5	Mid-swelling	Grape berries up to pea size (transverse diameter about 7 mm)	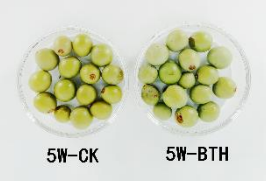
16 July 2022	7	End-swelling	Berries begin to soften and TSS begins to rise	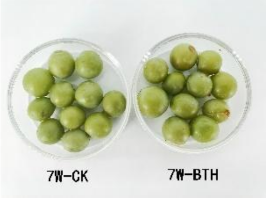
31 July 2022	9	Mid-veraison	50% grapes color change and softening, first rapid increase in weight	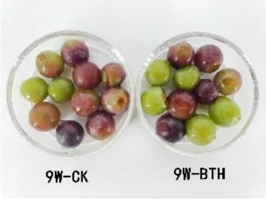
15 August 2022	11	End-veraison	grapes color change is completed and a second rapid increase in weight is accomplished.	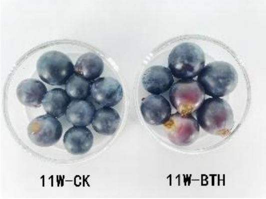
31 August 2022	13	Mid-ripening	grapes reached medium maturity (reducing sugar: 192.33 g/L, total soluble solids: 20.67 °Brix)	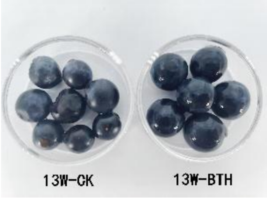
14 September 2022	15	Ripe	grapes with the highest sugar-acid ratio and technological maturity for harvest	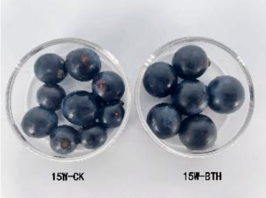

Table S2. Standard curve for quantification of C6 compounds, linolenic and linoleic

CAS	Compounds name	equation	R^2
6728-26-3	E-2-Hexenal	y = 2E-07x - 11.13	0.997 3
3681-71-8	Z-3-Hexenyl acetate	y = 3E-08x - 0.6 939	0.999 6
928-96-1	Z-3-Hexenol	y = 4E-07x - 3.4 715	0.997 4
111-27-3	Hexanol	y = 2E-07x - 2.4 623	0.991 6
66-25-1	Hexanal	y = 1E-07x - 75.367	0.994 0
142-92-7	Hexyl acetate	y = 4E-08x + 0.0 988	0.999 6
6789-80-6	Z-3-Hexanal	y = 2E-08x - 5.5 961	0.999 8
928-95-0	E-2-Hexenol	y = 3E-07x - 1.5 373	0.997 2
60-33-3	linoleic	y = 468.57x + 1069.1	0.999 1
463-40-1	linolenic	y = 479.41x + 1981.3	0.999 5

C6 compounds were detected by GC-MS, but linoleic and linolenic acid were detected by GC-FID.

Table S3. Concentrations of C6 compounds of BTH-treated (BTH) and CK grapes during ripening.

(a) Concentrations of free C6 compounds of BTH-treated and CK Cabernet Gernischt grapes (μg/L)

sample	(F)Hexanal	(F)E-2-Hexenal	(F)Z-3-Hexenal	(F) Hexanol	(F)Z-3-Hexenol	(F)E-2-Hexenol	(F)Hexyl Acetate	(F)Z-3-Hexenyl Acetate
15WCK1	546.946	3687.217	6.292	288.221	16.134	105.350	0.509	0.380
15WCK2	606.455	3692.040	6.446	340.586	21.280	102.176	0.526	0.357
15WCK3	504.764	3651.035	7.023	321.863	15.652	94.213	0.480	0.340
13WCK1	610.213	3781.818	5.332	261.114	54.469	39.633	0.497	0.544
13WCK2	598.645	3856.069	7.160	245.429	54.625	39.855	0.542	0.555
13WCK3	601.730	3699.636	7.599	235.401	56.541	35.892	0.526	0.568
11WCK1	328.809	3520.270	7.457	55.808	33.158	47.187	0.165	0.632
11WCK2	420.660	3696.375	6.946	65.094	38.482	47.316	0.140	0.745
11WCK3	391.822	3645.976	7.225	58.009	39.270	38.160	0.159	0.573
9WCK1	146.614	3119.654	2.478	62.887	99.256	33.967	1.073	3.531
9WCK2	140.669	2906.915	1.863	57.436	139.689	28.094	1.015	2.954
9WCK3	201.793	2775.983	2.282	43.255	130.008	30.414	1.109	3.447
7WCK1	171.629	1815.015	8.792	0.925	12.611	0.535	0.352	1.404
7WCK2	169.079	1992.199	10.846	1.191	13.082	0.405	0.231	1.350
7WCK3	177.154	1971.862	6.734	0.487	11.253	0.284	0.218	1.682
5WCK1	338.020	2064.480	12.320	5.170	31.070	0.510	0.960	3.710
5WCK2	346.180	2011.150	13.800	4.710	32.710	0.450	0.880	3.680
5WCK3	369.810	2079.260	11.880	4.300	32.350	0.480	0.960	4.890
3WCK1	413.187	1817.823	9.017	4.717	28.279	0.621	0.648	13.323
3WCK2	377.446	1813.943	11.408	6.066	27.378	0.596	0.452	11.858
3WCK3	413.894	2055.990	9.997	2.866	20.484	0.444	0.560	14.603
15WBTH1	493.076	3076.115	11.188	196.118	33.109	60.494	1.479	0.929
15WBTH2	495.694	3034.464	12.063	232.976	31.122	58.270	1.549	0.953
15WBTH3	456.429	3176.478	13.244	203.688	31.449	72.116	1.404	0.970
13WBTH1	544.588	3866.604	3.163	199.896	35.684	31.794	0.323	0.402
13WBTH2	508.113	3490.822	4.605	187.648	35.754	27.712	0.437	0.434
13WBTH3	469.963	3762.450	4.415	171.512	35.601	25.753	0.369	0.413
11WBTH1	384.437	3613.405	6.160	190.612	88.102	35.308	0.492	1.366
11WBTH2	348.226	3776.127	5.337	211.702	72.659	35.585	0.682	0.967
11WBTH3	347.099	3892.573	6.984	182.606	80.114	38.592	0.571	1.241
9WBTH1	226.498	2890.695	1.380	108.281	139.747	51.343	1.284	3.502
9WBTH2	205.886	2955.003	1.718	100.543	135.020	48.684	1.160	3.273
9WBTH3	184.764	2919.209	1.551	133.595	139.747	40.123	1.426	2.993
7WBTH1	262.711	2557.841	7.902	3.916	16.725	0.825	0.843	3.328
7WBTH2	275.352	2565.093	7.728	2.485	17.810	0.948	0.799	2.262
7WBTH3	250.836	2495.760	6.114	3.927	16.725	0.876	0.831	2.606
5WBTH1	267.420	2127.380	12.750	4.760	51.960	0.450	0.850	10.230
5WBTH2	337.600	2142.260	13.780	6.550	52.650	0.480	0.980	11.240
5WBTH3	335.800	1979.130	11.720	8.810	68.710	0.460	0.860	8.630
3WBTH1	420.354	1911.648	9.462	3.943	26.717	1.035	0.479	12.926
3WBTH2	398.655	1842.739	8.447	3.379	24.326	1.257	0.450	13.430
3WBTH3	381.403	1801.866	7.684	4.838	23.423	1.370	0.609	14.279

(b) Concentrations of bound C6 compounds of BTH-treated and CK Cabernet Gernischt grapes (μg/L)

sample	(B)Hexanal	(B)E-2-Hexenal	(B)Z-3-Hexenal	(B) Hexanol	(B)Z-3-Hexenol	(B)E-2-Hexenol	(B)Hexyl Acetat	(B)Z-3-Hexenyl

							e	Acetat e
15WCK1	72.982	3.165	6.502	17.254	4.655	1.943	0.416	0.083
15WCK2	70.027	3.215	6.641	15.769	4.229	1.618	0.357	0.111
15WCK3	71.850	3.296	6.479	18.398	4.457	1.889	0.357	0.095
13WCK1	75.773	20.430	8.097	18.565	10.994	3.154	0.347	0.056
13WCK2	74.889	20.627	8.551	17.067	10.290	2.844	0.360	0.069
13WCK3	75.150	19.723	8.188	21.144	10.399	2.934	0.370	0.044
11WCK1	76.769	55.602	5.951	15.583	12.461	0.400	0.420	0.176
11WCK2	76.823	54.045	5.898	16.706	12.794	0.390	0.398	0.147
11WCK3	77.645	54.854	6.073	14.374	12.120	0.382	0.391	0.174
9WCK1	81.167	93.767	8.691	8.906	2.861	0.206	0.280	0.045
9WCK2	80.857	93.483	8.086	6.763	2.716	0.195	0.288	0.051
9WCK3	80.404	99.521	8.311	7.364	2.891	0.181	0.292	0.049
7WCK1	38.366	52.274	10.945	0.943	1.199	2.674	0.338	0.053
7WCK2	38.284	52.517	11.572	1.169	1.183	2.397	0.366	0.049
7WCK3	38.026	52.401	10.630	1.375	1.189	2.646	0.362	0.055
5WCK1	28.290	21.167	8.245	0.396	0.746	1.489	0.329	0.157
5WCK2	28.265	18.307	7.894	0.353	0.869	1.467	0.318	0.160
5WCK3	28.264	17.853	7.920	0.367	0.763	1.475	0.337	0.150
3WCK1	19.157	12.144	9.286	3.472	0.689	2.498	0.364	0.052
3WCK2	19.266	12.116	9.518	3.448	0.660	2.665	0.305	0.056
3WCK3	19.335	12.204	9.655	3.222	0.604	2.465	0.352	0.059
15WBTH1	67.686	7.378	7.051	15.502	4.042	2.188	0.423	0.727
15WBTH2	67.995	7.306	7.399	14.747	4.180	1.455	0.441	0.674
15WBTH3	66.777	7.651	7.670	13.090	4.863	1.798	0.460	0.715
13WBTH1	72.628	22.498	8.925	6.601	6.196	1.514	0.408	0.274
13WBTH2	72.495	24.233	8.722	7.558	5.801	1.456	0.407	0.271
13WBTH3	72.218	23.756	8.359	8.045	5.480	1.386	0.409	0.281
11WBTH1	58.970	71.396	6.465	4.353	1.905	0.106	0.455	0.361
11WBTH2	56.479	71.173	6.224	4.240	2.683	0.116	0.470	0.414
11WBTH3	57.637	74.023	6.396	4.650	2.208	0.120	0.438	0.386
9WBTH1	51.087	44.474	8.852	1.608	1.973	0.134	0.343	0.223
9WBTH2	52.864	41.954	8.697	2.997	1.843	0.133	0.363	0.236
9WBTH3	51.005	48.107	8.927	2.163	1.834	0.140	0.381	0.227
7WBTH1	35.645	37.813	10.620	0.772	0.764	1.940	0.387	0.085
7WBTH2	35.673	37.485	10.705	0.731	0.720	1.814	0.361	0.082
7WBTH3	35.683	38.621	9.952	0.782	0.753	2.471	0.354	0.084
5WBTH1	25.645	15.457	8.230	0.417	0.336	1.559	0.311	0.125
5WBTH2	25.890	17.310	8.330	0.385	0.358	1.655	0.320	0.132
5WBTH3	26.030	19.108	8.334	0.405	0.323	1.793	0.329	0.129
3WBTH1	19.327	12.204	9.651	3.168	0.643	2.619	0.322	0.059
3WBTH2	19.305	12.264	9.898	3.230	0.705	2.522	0.338	0.061
3WBTH3	19.301	12.341	9.923	3.260	0.645	2.412	0.333	0.061

"(F)" and "(B)" indicate free and bound aromas. Sample names with suffixes 1,2,3 are three measurements of the same sample in order to show that the error is small.

Table S4. Correlation coefficients (rho) and p values for correlation analysis of linolenic acid, linoleic acid, enzyme activity, and C6 compounds

(a) Pearson correlation analysis during ripening of CK grapes

Data 1	Data 2	rho	*P*-value	relation
(F)C6 Esters	Linoleic acid	-0.660067	0.001129549	negative
(F)C6 Aldehydes	(F)C6 Esters	-0.5856642	0.005278257	negative
(F)C6 Alcohols	(F)C6 Esters	-0.5101305	0.01814514	negative
(B)C6 Alcohols	(F)C6 Esters	-0.5603716	0.008237413	negative
(F)C6 Esters	LOX	-0.5688032	0.007128845	negative
(F)C6 Esters	ADH	-0.5534165	0.009254958	negative
(F)C6 Compounds	(F)C6 Esters	-0.5814674	0.005696694	negative
(B)C6 Compounds	(F)C6 Esters	-0.5676249	0.007275934	negative
(F)C6 Aldehydes	Linoleic acid	0.8702644	2.93716E-07	positive
(F)C6 Alcohols	Linoleic acid	0.8846648	1.01825E-07	positive
(B)C6 Alcohols	Linoleic acid	0.7510957	8.71128E-05	positive
Linoleic acid	LOX	0.7217497	0.000221256	positive
ADH	Linoleic acid	0.7209444	0.00022662	positive
AAT	Linoleic acid	0.6311582	0.002153432	positive
(F)C6 Compounds	Linoleic acid	0.8860437	9.13375E-08	positive
(F)C6 Alcohols	(F)C6 Aldehydes	0.8701055	2.96961E-07	positive
(B)C6 Alcohols	(F)C6 Aldehydes	0.9493518	5.30275E-11	positive
(F)C6 Aldehydes	LOX	0.8293061	3.3648E-06	positive
(F)C6 Aldehydes	HPL	0.5598161	0.008315154	positive
(F)C6 Aldehydes	ADH	0.9206594	3.36582E-09	positive
(F)C6 Aldehydes	(F)C6 Compounds	0.9973804	3.85038E-23	positive
(B)C6 Compounds	(F)C6 Aldehydes	0.5075536	0.01883788	positive
(B)C6 Alcohols	(F)C6 Alcohols	0.7394091	0.000128105	positive
(F)C6 Alcohols	LOX	0.5849563	0.005346993	positive
(F)C6 Alcohols	ADH	0.6645922	0.001014826	positive
(F)C6 Alcohols	(F)C6 Compounds	0.9034386	2.03076E-08	positive
(B)C6 Aldehydes	(B)C6 Compounds	0.9759964	4.88615E-14	positive
(B)C6 Alcohols	LOX	0.8844959	1.0318E-07	positive
(B)C6 Alcohols	HPL	0.5816976	0.00567305	positive
(B)C6 Alcohols	ADH	0.972406	1.81147E-13	positive
(B)C6 Alcohols	(F)C6 Compounds	0.9341477	6.047E-10	positive
(B)C6 Esters	HPL	0.7270622	0.000188528	positive
ADH	LOX	0.8987675	3.12193E-08	positive
(F)C6 Compounds	LOX	0.8067565	9.95716E-06	positive
ADH	HPL	0.6322229	0.002105232	positive
(F)C6 Compounds	HPL	0.5232622	0.01492588	positive
(F)C6 Compounds	ADH	0.8981799	3.29061E-08	positive

(b) Pearson correlation analysis during ripening of BTH grapes

Data 1	Data 2	rho	*P*-value	relation
(F)C6 Aldehydes	Linolenic acid	-0.6340386	0.00202512	negative
(B)C6 Alcohols	Linolenic acid	-0.507487	0.01885605	negative
Linolenic acid	LOX	-0.7203229	0.000230836	negative
ADH	Linolenic acid	-0.679843	0.000697981	negative
(F)C6 Compounds	Linolenic acid	-0.6173416	0.002867966	negative

(F)C6 Aldehydes	(F)C6 Esters	-0.8670923	3.64744E-07	negative
(F)C6 Alcohols	(F)C6 Esters	-0.6983127	0.0004306	negative
(B)C6 Aldehydes	(F)C6 Esters	-0.8486462	1.16265E-06	negative
(B)C6 Esters	(F)C6 Esters	-0.6396707	0.001792743	negative
(F)C6 Esters	LOX	-0.6925931	0.000501921	negative
(F)C6 Esters	ADH	-0.6997299	0.000414334	negative
(F)C6 Esters	AAT	-0.5169859	0.01640141	negative
(F)C6 Compounds	(F)C6 Esters	-0.8620836	5.07862E-07	negative
(B)C6 Compounds	(F)C6 Esters	-0.8989962	3.05838E-08	negative
Linoleic acid	Linolenic acid	0.6963893	0.000453548	positive
(F)C6 Alcohols	(F)C6 Aldehydes	0.8057354	1.04238E-05	positive
(B)C6 Aldehydes	(F)C6 Aldehydes	0.8501581	1.06346E-06	positive
(B)C6 Alcohols	(F)C6 Aldehydes	0.5754799	0.006340839	positive
(B)C6 Esters	(F)C6 Aldehydes	0.6943538	0.000478965	positive
(F)C6 Aldehydes	LOX	0.9323833	7.71962E-10	positive
(F)C6 Aldehydes	HPL	0.5996719	0.004061339	positive
(F)C6 Aldehydes	ADH	0.9075363	1.36733E-08	positive
(F)C6 Aldehydes	AAT	0.5010399	0.02068386	positive
(F)C6 Aldehydes	(F)C6 Compounds	0.9958937	2.73861E-21	positive
(B)C6 Compounds	(F)C6 Aldehydes	0.9202051	3.54701E-09	positive
(B)C6 Aldehydes	(F)C6 Alcohols	0.7651044	5.33447E-05	positive
(B)C6 Alcohols	(F)C6 Alcohols	0.5424106	0.0110736	positive
(B)C6 Esters	(F)C6 Alcohols	0.7924768	1.84609E-05	positive
(F)C6 Alcohols	LOX	0.7901252	2.03443E-05	positive
(F)C6 Alcohols	HPL	0.7145951	0.000272969	positive
(F)C6 Alcohols	ADH	0.8545171	8.17855E-07	positive
(F)C6 Alcohols	(F)C6 Compounds	0.8560198	7.45609E-07	positive
(B)C6 Compounds	(F)C6 Alcohols	0.834224	2.60121E-06	positive
(B)C6 Aldehydes	LOX	0.7669469	4.98895E-05	positive
(B)C6 Aldehydes	HPL	0.6433671	0.001652783	positive
(B)C6 Aldehydes	ADH	0.8544616	8.20637E-07	positive
(B)C6 Aldehydes	(F)C6 Compounds	0.8582767	6.47641E-07	positive
(B)C6 Aldehydes	(B)C6 Compounds	0.9804201	7.17812E-15	positive
(B)C6 Alcohols	(B)C6 Esters	0.8154773	6.65914E-06	positive
(B)C6 Alcohols	LOX	0.5587855	0.00846097	positive
(B)C6 Alcohols	(F)C6 Compounds	0.5852788	0.005315589	positive
(B)C6 Esters	LOX	0.6719551	0.000849291	positive
(B)C6 Esters	HPL	0.6853747	0.000606129	positive
(B)C6 Esters	ADH	0.6100467	0.003319023	positive
(B)C6 Esters	(F)C6 Compounds	0.7268888	0.000189526	positive
(B)C6 Compounds	(B)C6 Esters	0.6302127	0.002197014	positive
HPL	LOX	0.6940879	0.000482372	positive
ADH	LOX	0.90477	1.78933E-08	positive
(F)C6 Compounds	LOX	0.9351454	5.25126E-10	positive
(B)C6 Compounds	LOX	0.8381282	2.10787E-06	positive
ADH	HPL	0.730001	0.000172282	positive
(F)C6 Compounds	HPL	0.6332541	0.002059412	positive

(B)C6 Compounds	HPL	0.6751878	0.000784216	positive
(F)C6 Compounds	ADH	0.9231348	2.51529E-09	positive
(B)C6 Compounds	ADH	0.8970335	3.6431E-08	positive
(B)C6 Compounds	(F)C6 Compounds	0.9300318	1.05827E-09	positive

“ (F)” and “(B)” indicate free and bound aromas. Analyzed data downloaded from https://www.omicstudio.cn (accessed on 4 September 2022).
